# Vocational Bias: A Potential Pitfall in the Use of Key Informant Interviews in Pediatric Community Needs Assessments

**DOI:** 10.1100/tsw.2008.72

**Published:** 2008-04-29

**Authors:** Kimberly Northrip, Candice Chen, Jennifer Marsh

**Affiliations:** ^1^ Department of Pediatrics, University of Kentucky, 740 South Limestone St., Lexington, KY 40536, USA; ^2^ Department of General Pediatrics, Children's National Medical Center, 111 Michigan Ave., NW, Washington, D.C. 20010, USA; ^3^ Office of Medical Services, Peace Corps Headquarters, 1111 20^th^ St., NW, Washington, D.C., USA

**Keywords:** key informant, needs assessment, community pediatrics

## Abstract

Key informants are individuals with insight into a community or a problem of interest. Our objective was to evaluate the effect of the employment type of key informants on the outcome of a pediatric needs assessment for an urban community. Twenty-one interviews were conducted during the course of a pediatric community needs assessment. As part of the interview, informants were asked to list the top three problems facing children in their community. We analyzed their answers to determine if informant responses differed by employment type. Key informants were divided into four employment types: health care setting, social service, business, and infrastructure. Responses were coded as being primarily one of three types: medical, social, or resource. Our results showed that those informants who worked in a health care setting listed medical problems more often than those who did not (p < 0.04). Those who worked in social services listed resource problems more often than those who did not (p < 0.05). Those in business and infrastructure positions listed more social problems (p < 0.37). No difference was observed in response type between those who had lived in the community at some point and those who had not. This study lends support to the hypothesis that informants’ reporting of community problems is biased by their vocation. Clinicians often focus their needs assessments on health care workers. This study suggests, however, that we need to take into consideration the bias this presents and to seek to interview people with diverse work experiences. By limiting the process to health care workers, clinicians are likely to get a skewed perspective of a community’s needs and wants.

## INTRODUCTION

A key informant (KI) is someone who has special knowledge about the topic or population of interest, possibly as a result of their social position or skills[1,2]. For example, business and civic leaders are often able to provide unique insights into their communities that health care providers may not see.

The technique of interviewing KIs was first developed in the field of cultural anthropology in order to learn more about unfamiliar societies. This technique has become a fundamental research process and is currently used in many areas, including medicine, organizational research, and public health[1,3]. For example, a Medline search using the keyword “key informants” yielded over 550 articles, many of which used KI interviews to obtain information about a given need, a disease within a population, or attitudes regarding a health program[4].

As with many data collection techniques, KI interviews have advantages and disadvantages. These interviews are cost effective, require little time, and can yield valuable, in-depth qualitative information[5]. However, KIs may be biased, they may not represent the majority view, they may be unwilling to reveal sensitive or politically incorrect information, or, on rare occasion, they may outright lie[1,6].

The organizational research literature has documented several different types of KI biases, including position bias, rater-trait interaction bias, recall bias, and response bias[3,5]. Position bias is bias created by the role a person plays in an organization. A person’s role or rank may affect the kind of information they have access to and may affect how honest KIs are willing to be with the interviewer due to fear of displeasing superiors[3]. Rater-trait interaction bias refers to biases caused by traits specific to the KI other than position or role[3]. These might include age, gender, personality traits, etc. For example, a study of Catholic clergy found that older clergy were less likely to have negative attitudes about their superiors than younger clergy [7].

Recall bias is caused by an informant’s ability to remember facts. More complex questions increase the opportunity for recall bias. For example, questions requiring a KI to draw conclusions about abstract issues will increase the chances of recall bias[3]. Lastly, response bias may result from KI factors affecting their willingness to participate. For example, when trying to get an idea of what changes need to be made in a community, you may hear more from those individuals who are unhappy than those who enjoy living there[5]. These forms of bias deal with attributes of individuals not exclusive to large organizations. It is logical, therefore, that these forms of bias will also affect KIs used for other purposes, such as community needs assessments.

None of these forms of bias directly address the KI’s vocation. Vocation may be less relevant to KI work done within more homogeneous organizations, however, communities generally incorporate many diverse individuals and as KI interviews have become an important tool in community needs assessments[8], vocational differences become more relevant. Therefore, this study seeks to determine if vocation is a potential source of bias for KI interviews.

## METHODS

The objective of this study was to determine whether a KI’s type of employment would bias the outcome of a pediatric needs assessment for an urban community. Twenty-one KI interviews were conducted during the course of a pediatric community needs assessment in the poor African American community of Washington, D.C. called Ward 8 from February through June of 2005. As part of the interview, informants were asked to identify the top three problems facing children in their community. We analyzed these data to determine if informant responses differed by employment type. Interviewers were divided into four employment types: health care setting, social service, business, and infrastructure. They were also asked if they had ever lived in Ward 8 (see Table 1).

Responses to the question regarding the top three problems facing children were coded as being primarily one of three types: medical, social, or resource (see Table 2). Response types were then examined compared to employment type and history of residence in the community. One participant was unemployed and was omitted from the vocational analysis. Fisher’s Exact test was used to determine statistical significance.

**TABLE 1**
**Jobs by Category**

| |
|---|
| Health Care Setting |
| Clerks |
| Nurses |
| Health educator |
| School nurse |
| Methadone clinic staff |
| Pharmacist |
| Social Services |
| Women, infants, and children employees |
| Charity organization workers |
| Business |
| Grocery store workers and manager |
| Liquor store clerk |
| Fast food restaurant manager |
| Infrastructure |
| School and church daycare providers |
| Church staff |
| Teachers |
| Police |

**TABLE 2**
**Response Category Definitions and Contents**

| | |
|---|---|
| **Medical** | Fits a strict diagnosis or symptom, may have social or resource influences. Examples: asthma, teen pregnancy, HIV/STDs, delayed immunizations, poor nutrition, drug and alcohol addiction. |
| **Social** | Behaviors or conditions that may affect health, but are not individual diagnoses. Examples: early sexual activity, high rates of foster care, violence, crime, lack of role models and guidance, difficulty accessing services, parenting skills, trust between community and health care providers, literacy, unemployment, health literacy. |
| **Resource** | Tangible things that members of the community think are inadequate. Examples: poor schools, need for more services to single parents, poor housing, too many programs for certain problems and not for others, after school programs, affordable daycare, need for emergency funding, activities for kids. |

## RESULTS

Informants who worked in a health care setting listed medical problems more often than those who did not ($p < 0.04$). Informants who worked in a social service setting listed resource problems more often than those who did not ($p < 0.05$). Those working in business and infrastructure positions listed more social problems, but the difference was not statistically significant ($p < 0.37$). No difference was observed in response type between those who had lived in the community at some point and those who had not (see Table 3).

**TABLE 3**
**Problem Type by Job Category**

| Top Three Problems | Health Care | Social Service | Business | Infrastructure |
|---|---|---|---|---|
| Resource problems | 22.33% | 55.67% | 16.67% | 16.67% |
| Social problems | 33.33% | 44.33% | 58.33% | 58.33% |
| Medical problems | 44.33% | 0.00% | 25.00% | 25.00% |
| n = 21 | 9 | 3 | 4 | 4 |

## DISCUSSION

KIs are used with increasing frequency in health care research. Organizational research describes multiple forms of KI bias, however, health care research and community needs assessments are unique, often involving diverse stakeholders with different experiences and interests. Very little research has been done on these differences. In this study, we examined the role of vocational bias, which differs from the previously mentioned forms of bias. Vocational bias may be similar to other forms of bias because the profession of an individual will affect what information they may have and what they might offer when asked. However, vocational choice does not always directly relate to position within an organization. For example, the differences in the insights of the clerk at the police station and the chief of police could be explained by position bias. However, how those insights differ from those of someone in an entirely different field, but of equivalent rank, could not. This type of bias, vocational bias, was demonstrated here in KI interviews of individuals from diverse “ranks” within four employment types during the course of a community needs assessment.

Informants in the field of health care had a significant tendency to cite medical problems as the most important in a community, while those in social service positions demonstrated a preference for resource problems. The study did not find a significant difference in problem selection for those in business and infrastructure fields.

These findings suggest that priorities for a community will differ among those in different vocational groups. This may be because of the personal traits and preferences that led an individual to select a job initially and/or because of the way an individual interacts with the community through the duties of a given employment type. The community’s needs most commonly witnessed during the execution of work duties differ from vocation to vocation; therefore, a health care worker, for example, would give medical problems the highest priority because those are the problems most commonly witnessed by that individual. Additionally, the health care worker possibly chose that vocation due to pre-existing perceptions of the importance of the duties carried out by the profession. Similar statements could be made about the other three employment types.

This has major implications for using KIs in health care research and community needs assessments. Community needs assessments that interview KIs from predominantly one or two employment types are likely to get a skewed view of the community and its problems. Vocational bias may have added significance because vocation is often used as a criterion for KI selection. Marc-Adelard Tremblay (see Marshall[1]) identified five characteristics of a good key informant: role in community, knowledge, willingness, communicability, and impartiality. Of these characteristics, an interviewer can be sure only of the KIs role initially. Therefore, we are likely to begin by identifying potential KIs by their community role and then attempt to determine if they meet the other criteria before conducting an interview[1]. One straightforward way of classifying a KI’s role in a community is by job description. Other factors may also play a part in identifying role, such as reputation as a community leader, but vocation is likely to be strongly considered as it is easy for the investigator to identify and has implications for community knowledge.

Because a KI may be selected due to social roles and types of employment believed to imply familiarity with the community and problem of interest, the potential for vocational bias is amplified. The risk of bias can be minimized by increasing the diversity of KIs interviewed with relationship to employment type. For example, a social services organization interested in examining the needs of the homeless in a city may focus its search for KIs in the local shelters and food banks — both social service employment types. This would increase the chance of bias. The homeless also interact with those in other vocational types. It may be possible to get a more complete and less biased view of the homeless community by also including KIs who work in medical centers, public transportation, and businesses serving low income and homeless clientele. As researchers and community physicians, we should be cautious not to allow our own biases to lead us to seek KIs within our own fields, who give us familiar and comfortable answers to our questions, but instead seek KIs from a range of vocations capable of creating a more complete picture of the community of interest.

This study had a number of limitations. Participant numbers were low, which affects the study's power to detect statistically significant differences. The study was also a retrospective look at pre-existing data. KIs were selected by a KI tree, not by vocational group, and the job categories were retrospectively applied. This caused group sizes to vary widely. A larger prospective study with a more equitable distribution of participants from each vocational type would lend further evidence to the existence of vocational bias in KI interviews.

Despite its limitations, results of the study supported the hypothesis that informants' reporting of community problems is biased by their vocation in that community. Clinicians and researchers who seek to understand the needs of their community more completely should interview people with diverse work experiences in order to avoid this bias. By limiting the process to health care workers, clinicians may get a biased perspective of the community's needs and wants.
